# Preparing for an Australian Football League Women's League Season

**DOI:** 10.3389/fspor.2020.608939

**Published:** 2020-12-23

**Authors:** Heidi Rose Thornton, Cameron R. Armstrong, Alex Rigby, Clare L. Minahan, Rich D. Johnston, Grant Malcolm Duthie

**Affiliations:** ^1^Gold Coast Suns Football Club, Metricon Stadium, Carrara, QLD, Australia; ^2^Griffith Sports Science, Griffith University, Gold Coast, QLD, Australia; ^3^School of Behavioural and Health Sciences, Australian Catholic University, Brisbane, QLD, Australia; ^4^School of Exercise Science, Australian Catholic University, Strathfield, NSW, Australia

**Keywords:** acceleration, speed, team sport, GPS, intensity

## Abstract

The aims were to investigate the externally measured weekly loads, and the distribution intensity relative to the 1-min maximal mean (MM) intensity of matches. Athletes (*n* = 28) wore 10 Hz GNSS devices during training and matches. For the descriptive analysis, a range of movement variables were collected, including total distance, high-speed distance, very high-speed distance, acceleration, and acceleration load. Using raw GNSS files, 1-min moving averages were calculated for speed (m·min^−1^) and acceleration (m·s^−2^), and were multiplied by time, specifying total distance (m), and by body mass to quantify impulse (kN·s^−1^). The distribution of distance and impulse accumulated at varied intensities relative to MMs was calculated, with percentages ranging from zero to 110%. Drills were categorized as either; warm-ups, skill drills, games (i.e., small-sided games), conditioning and matches. Linear mixed models determined if the distribution of intensity within each threshold (>50%) varied between drill types and matches, and if the distribution within drill types varied across the season. Effects were described using standardized effect sizes (ES) and 90% confidence limits (CL). Compared to matches, a higher proportion of distance was accumulated at 50% of the MM within warm-ups and conditioning (ES range 0.86–1.14). During matches a higher proportion of distance was accumulated at 60% of MM when compared to warms ups, skill drills and conditioning (0.73–1.87). Similarly, greater proportion of distance was accumulated between 70 and 100% MM in matches compared to skill drills and warm-ups (1.05–3.93). For impulse, matches had a higher proportion between 60 and 80% of the MM compared to conditioning drills (0.91–3.23). There were no other substantial differences in the proportion of impulse between matches and drill types. When comparing phases, during competition there was a higher proportion of distance accumulated at 50% MM than general preparation (1.08). A higher proportion of distance was covered at higher intensities within matches compared to drills. The proportion of impulse was higher between 60 and 80% MM within matches compared to conditioning. Practitioners can therefore ensure athletes are not only exposed to the intensities common within competition, but also the volume accumulated is comparable, which may have positive performance outcomes, but is also extremely important in the return to play process.

## Introduction

The Australian Football League Women's (AFLW) is a national, two-conference competition comprizing 14 teams across five states of Australia. The AFLW has expanded since its inaugural season in 2017 by increasing the number of teams in the competition and the number of games played in a season–which has attracted more support, funding and ultimately professionalism to the sport. AFLW has similar playing rules to the men's Australian Football League (AFL) competition, with the main purpose of advancing the ball down the field by either kicking or “handballing” the ball and scoring points by kicking the ball between the upright posts (Robertson et al., 2016; Johnston et al., 2018). Although there are some modifications compared to AFL (Clarke et al., 2018), AFLW can also be described as a high-intensity, intermittent team sport (Clarke et al., 2018; Thornton et al., 2020). Typically, AFLW athletes cover between ~5–7 km during each match, with ~50 min of playing time (Clarke et al., 2018; Thornton et al., 2020), equating to a mean running speed (m·min^−1^) of between 102 and 128 m·min^−1^. Running speed constantly changes during matches, resulting in a mean acceleration of 0.44 m·s^−2^ (Thornton et al., 2020), reflecting the importance appropriately training this capacity. Running efforts are interspersed with rest intervals (walking or standing still) and technical skills such as kicking, tackling, and marking. These data (Clarke et al., 2018, 2019) provide an understanding of the volume, intensity and type of locomotive activity covered in matches that can be useful in optimizing training prescriptions – an area of AFLW that has not yet been presented in the scientific literature.

As demonstrated across numerous team sports (Delaney et al., 2016, 2017,a; Duthie et al., 2019), assessing the mean intensity of competition does not provide accurate information regarding the most intense passages of play. If maximum playing intensity achieved during matches is not accounted for in the training plan, this may result in athletes not being optimally prepared for competition which may negatively impact athletes and potentially increase the risk of injury. Moving averages have been established as an effective and simple method to quantify fluctuations in intensity that occur during team-sport competition (Cunningham et al., 2018). Moving averages involve calculating the mean of a variable over a select period (i.e., 1 min), then forward shifting over the length of the dataset, where the maximum value of that period is then extracted (Johnston et al., 2020). This maximal mean (MM) value can be calculated across a range of movement variables, and regardless of the variable assessed, a consistent finding in research is an evident decline in intensity as the duration of the moving average increases (Delaney et al., 2016, 2017,a; Duthie et al., 2019; Thornton et al., 2020). Practically, MMs can be used as a guideline regarding the intensity of drills of differing duration with the purpose of exposing athletes to match intensity [i.e., during small-sided games (SSG)] (Duthie et al., 2019). Whilst MMs are extremely useful in the prescription of such training drills, it must be noted that the “peak” or maximal value attained throughout a match only occurs once, therefore not reflecting the overall fluctuating intensity of the match (Johnston et al., 2020). Indeed, one study showed little difference between professional and semi-professional rugby league competition in 1–10-min MMs; suggesting that these periods may not reflect the overall demands of competition (Johnston et al., 2019). Athletes may only reach this MM value or near this for 1-min of the game, therefore it may be unnecessary to expose athletes to this intensity for large volumes (Johnston et al., 2020), as per typical periodization principles. Further work is required to determine the quantity of work that is required at these intensities.

Recently, an alternative method of describing the intensity of competition has been investigated where the distribution of volume covered relative to the 1-min MM value for a range of variables was presented (Johnston et al., 2020). Specifically, the intensity accumulated relative to the MM value was expressed in 10% buckets (i.e., 110–100%, 100–90%, all the way to 0). The distribution (%) of volume was used rather than simply volume accumulated as this standardizes the variables assessed, and accounts for differing game time. In this research (Johnston et al., 2020), most match activities (quantified using total distance, accelerometer load, and impulse) were performed at ~60% of peak match intensity for both professional Australian football (i.e., AFL) and rugby league (i.e., NRL) (Johnston et al., 2020). Further, within AFL, for the three movement variables investigated, athletes accumulated 13% of total distance, 7% of total impulse, and 11% of the total accelerometer load above 70% of the 1-min MM (Johnston et al., 2020). Together, this information emphasizes that perhaps prescribing training simply by using the MM value may result in excessive volume covered at intensities that are not sustained for periods of time during competition (Johnston et al., 2020). As such, it would be useful for practitioners to understand the distribution of intensity within drills (particularly skill-based drills), providing information that would help prescribe training that accurately reflects the volume covered at various intensities of competition.

Therefore, the purpose of this investigation was to (a) provide an overview of the weekly externally measured training loads across the AFLW season, which will assist in the preparation of athletes for competition. The second component, part (b) was an analysis of the training undertaken, where the distribution of volume accumulated within training drills relative to the 1-min MM intensity of matches was established. This involved comparing the distribution of intensity of different drill types compared to that obtained within matches and each drill type from training sessions, and further determining if the distribution of intensity changed across the various season phases. Furthermore, the information regarding the intensity distribution of drills will help ensure practitioners are not only exposing athletes to the MM intensity of competition within training, but also are achieving comparable volumes across a range of intensities.

## Methods

### Design

To quantify the physical demands of AFLW training across the 2020 season, an observational longitudinal research design was used. Workload data were collected using global navigation satellite system (GNSS) technology during training. Written informed consent was provided prior to the commencement of the study, and institutional ethics approval were obtained from the Australian Catholic University Human Research Ethics Committee (HREC no; 2018-290E).

### Subjects

Data were collected from 28 athletes playing for one club competing in the AFLW 2020 season (age: 24.1 ± 4.9 y; mass: 68.3 ± 6.5 kg; height: 171.9 ± 6.7 cm). Although athletes were not separated into positional groups for the analysis, athletes were from all positional groups of the squad, including midfielders (*n* = 12), rucks (*n* = 1), mobile backs (*n* = 5), mobile forwards (*n* = 4), tall backs (*n* = 2), and tall forwards (*n* = 4).

### Training Program

A periodized game-specific training program was prescribed and completed at the discretion of coaching and performance staff. An overview of the typical pre-season and in-season weekly schedule is demonstrated in Table 1. Some weeks did not follow this structure [i.e., in-season some weeks involved a session the day before a match (match−1) rather than a match−2].

**Table 1:** Summary of a typical training week during the pre-season and in-season periods.

	**Monday**	**Tuesday**	**Wednesday**	**Thursday**	**Friday**	**Saturday**	**Sunday**
**Pre-season**							
Morning(5:30 a.m.−7:00 a.m.)	-	Gym	-	Gym	OFF	Field	OFF
Evening(5:00 p.m.−8:30 p.m.)	Field	-	Field	-	OFF	-	OFF
**In-season**							
Morning(5:30 a.m.−7:00 a.m.)	-	-	Gym	-	OFF		OFF
Evening(5:00 p.m.−8:30 p.m.)	Recovery (30 min)	Field	-	Field	OFF	Match	OFF

*The in-season week is a typical 7-day turnaround*.

*This table shows a “typical” week therefore some weeks training did not follow this structure. Some weeks included a session the day prior to a match (match−1) rather than 2 days prior (match−2)*.

The season was 17 weeks in duration, with the pre-season phase being over a 10-week period. Specifically, general preparation was between weeks 1 and 4, followed by a 1-week Christmas break. Specific preparation was between weeks 6 and 10, with 7 weeks of competition following. Files were removed if an athlete was unable to complete the session due to injury or other reasons, and if the session was modified compared to the remaining group (i.e., load management) as to not affect group loads. As such, there were a total of 1,081 observations (920 training sessions and 161 matches). The mean ± SD and range of observations for each athlete including training sessions and matches was 36 ± 11 (range 3 to 47).

### Descriptive Training Loads

During all training sessions and games, microtechnology devices (Optimeye S5, Catapult Sports, VIC, Australia) were used to measure the external workloads of athletes. These devices comprise a 10 Hz GNSS chip, and athletes wore the same device across the season as to minimize inter-unit variability (Buchheit et al., 2014). Prior to the start of training, units were switched on and were fitted into a manufacturer provided garment to tightly secure the device. Data quality was determined by recording the horizontal dilution of position (HDOP; mean ± SD = 0.68 ± 0.09) and satellite count (11.79 ± 0.75). Four files with a HDOP >1.5 were removed. Following training sessions and games, devices were downloaded and trimmed using proprietary software (Openfield, Catapult Sports, VIC, Australia). Numerous movement variables were obtained from the software, including; total distance (m), speed (m·min^−1^), high-speed distance (>14.4 km·h^−1^), very high-speed distance (>20 km·h^−1^), acceleration (m·s^−2^) and acceleration load (AU). The metrics chosen in this have been demonstrated as reliable in various studies using the same devices as well as previous models (Johnston et al., 2014; Delaney et al., 2017b; Weaving et al., 2017; Thornton et al., 2019).

### Intensity Analysis

In addition to using the generic export of the summary metrics as detailed below, following training, raw data (10 Hz) were exported to Microsoft Excel as comma separated value (csv) files. These files included details such as time and speed. Speed was adjusted to mmin^−1^ by multiplying by “60,” and acceleration was converted to positive values only, as in its original format it included negative values, reflecting decelerations (Delaney et al., 2017b). Moving averages were calculated over 1-min for speed and acceleration using customized software (RStudio v.1.1.383, RStudio, Boston, MA). A 1-min moving average was selected as this reflects the fluctuating intensity of team sports, although appropriately smooths the data to represent true changes in intensity. Further, as a 1-min MM value is used to represent the peak match intensity, using the same time period to determine the relative intensity of drills is deemed as being most appropriate. Maximal means from the same cohort of athletes has been previously established, where no positional differences were evident for both speed and acceleration, therefore, global values of speed (205 m·min^−1^) and acceleration (0.70 m·s^−2^) were used as reference values (Thornton et al., 2020). Following, the volume of speed [distance (m)] and acceleration [impulse (kN·s^−1^)] accumulated in 10% buckets (i.e., 110–100%, 100–90%, all the way to 0) was determined. As the MM value was a mean obtained from the group from all files (not simply an athletes' own within each match file), within matches some athletes were able to accumulate distance and impulse above the MM value.

**Figure 1:**
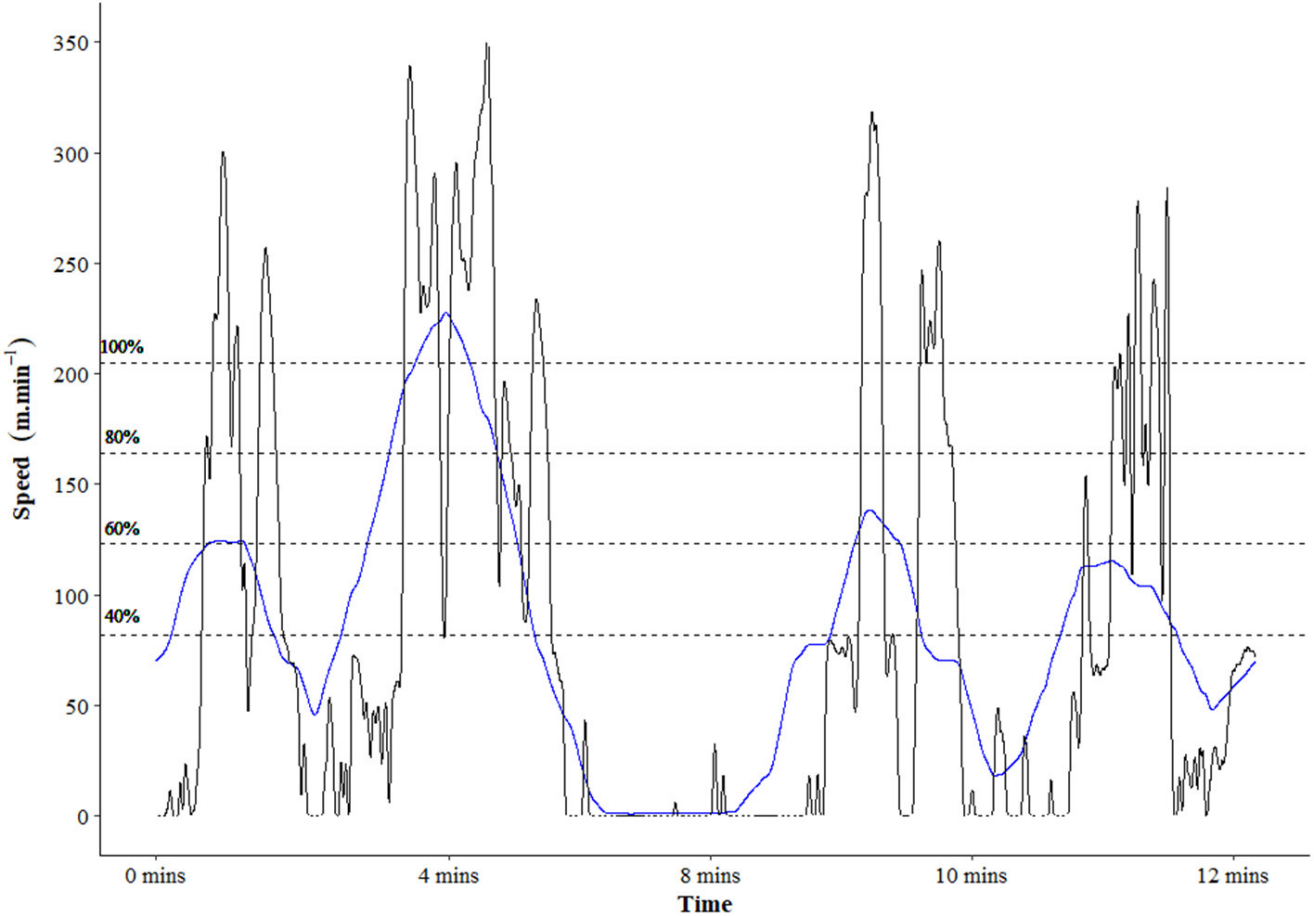
Example 1-min moving average of speed (blue line) and raw speed (black line) across a training game, demonstrating the fluctuating intensity and the purpose of this moving average method. The dotted lines represent values relative to the maximal mean (205 m·min^−1^) at 20% increments. NB. This file represents one athlete's match file, and in this match could reach speed above the reported maximal mean of 205 m·min^−1^ that is obtained from the squad.

Figure 1 demonstrates an example of a 1-min moving average for speed across a drill, as well as the raw speed to demonstrate the purpose of applying a moving average to such data. On this figure, the MM value is demonstrated and 20% buckets (starting at 40%). This figure demonstrates the fluctuating intensity across the drill, and volume can be accumulated at different intensities. For example, based on the 1-min MM speed of 205 m·min^−1^, 90–100% corresponds to speed between 185 and 205 m·min^−1^, and the distance accumulated at such intensity was calculated. To account for differing drill lengths, the total volume of distance and impulse covered within drill was calculated, and the volume within each bucket was then divided by the total volume, providing the percentage distribution.

### Drill Types

During each training session, drills were labeled according to its primary purpose, categorized as either warm up, skill drill, game (i.e., SSG or high-intensity game), conditioning or matches (official AFLW matches). Matches were also included as a “drill” allowing comparisons to be made with training drills. Specifically, within these categories, warm up represents any drill that is designed to prepare athletes for the session and includes warm up kicking drills. Skill drills include those which are designed to learn or focus on concepts such as kicking, tackling, and handballing. Games include drills used to replicate game scenarios such as match simulation, SSGs and other high-intensity concept drills. Conditioning includes drills solely focused on increasing aerobic power, anaerobic capacity, speed, agility, acceleration, and deceleration movements. Matches included each quarter of AFLW competition. For this analysis, sessions the day prior to the match (match−1) were removed (although most weeks did not have a match−1 session), as they typically involve low intensity craft, or individual skill, as were sessions 2 days post-match (match +2), as this is generally mobility and straight line, low-intensity running. Within the final dataset, there were a total of 800 warm up files, 2,400 skill drills, 1,161 games, 606 conditioning drills, and 523 matches.

### Statistical Analysis

Data were assessed for normal distribution using a Shapiro-Wilk test. There were no statistical comparisons made between season phases for the weekly externally measured training loads, as practically this is not deemed as useful. Linear mixed models were used to compare the intensity distribution of each drill type compared to matches within each bucket. In this model, the outcome variables were each intensity bucket, the fixed effect was drill type, and the random effect was the athlete identification. Further, within each drill type, the change in the distribution of intensity across each phase was similarly investigated using linear mixed models. Here, the outcome was each drill type separately (excluding matches as these were only played during competition), the fixed effect was the phase of the season, and the random effect was the athlete identification. Resulting SDs and mean differences were then assessed to establish standardized effect sizes (ES) and 90% confidence limits (CL), and ES were described using the magnitudes; <0.20 trivial; 0.21–0.60 small; 0.61–1.20 moderate; 1.21–2.0 large and >2.01 very large (Hopkins et al., 2009). Effects were deemed to be real if they were 75% greater than the smallest worthwhile difference (SWD; calculated as 0.6 x the between-athlete SD) (Hopkins et al., 2009) based on reasons explained in previous research (Duthie et al., 2019; Johnston et al., 2020). All statistical analyses were performed in R Studio software (version 1.3.959, RStudio Inc.).

## Results

**Figure 2:**
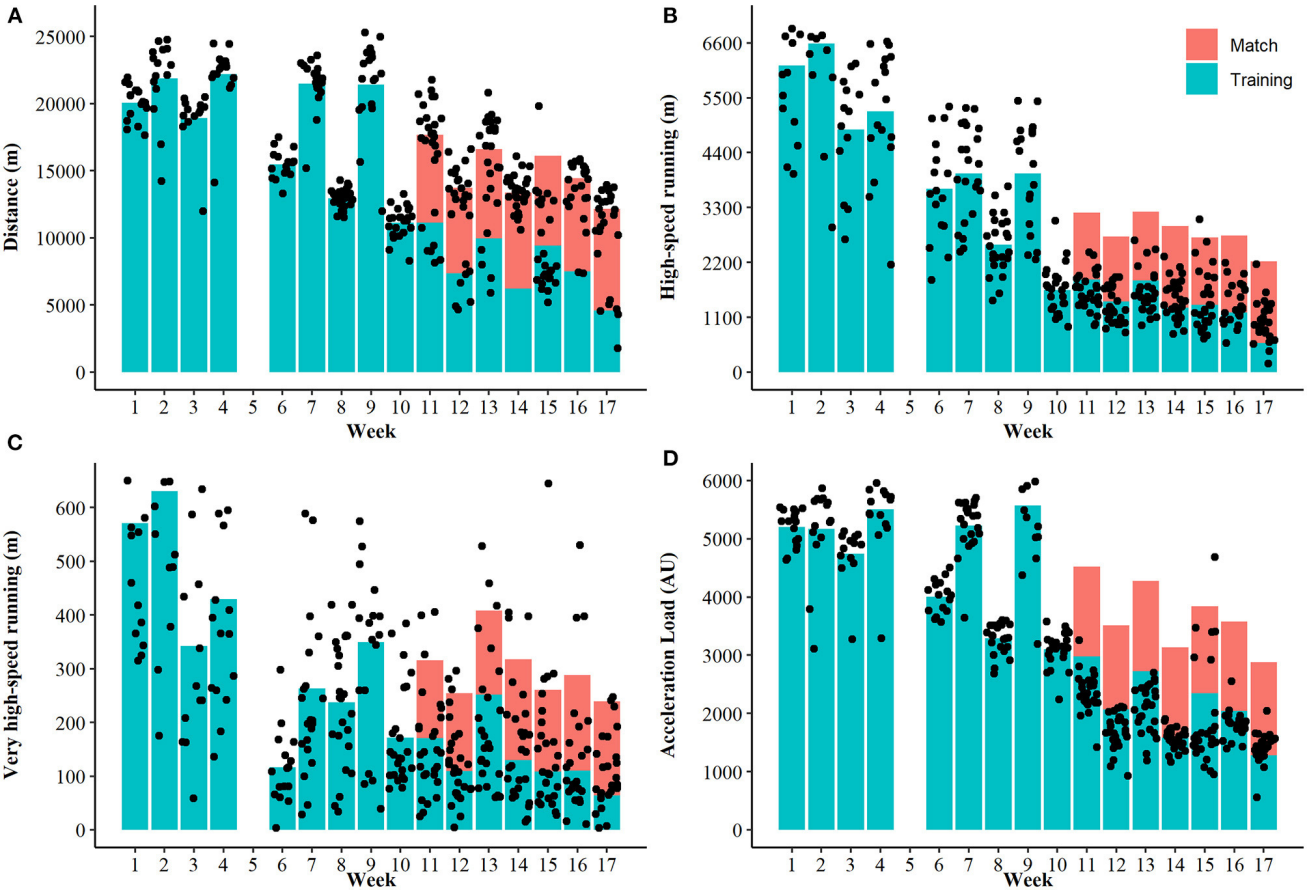
Mean (bars) and individual (dots) weekly physical demands of training and matches across the pre-season and in-season phases. The pre-season phase was week 1–10 (general preparation was weeks 1–4, specific preparation weeks 6–10), and competition was weeks 11–17 for **(A)** distance, **(B)** high-speed running, **(C)** very high speed running, and **(D)** acceleration load. *high-speed running is >14.4 km·h^−1^ and very-high speed running is >20 km·h^−1^.

**Figure 3:**
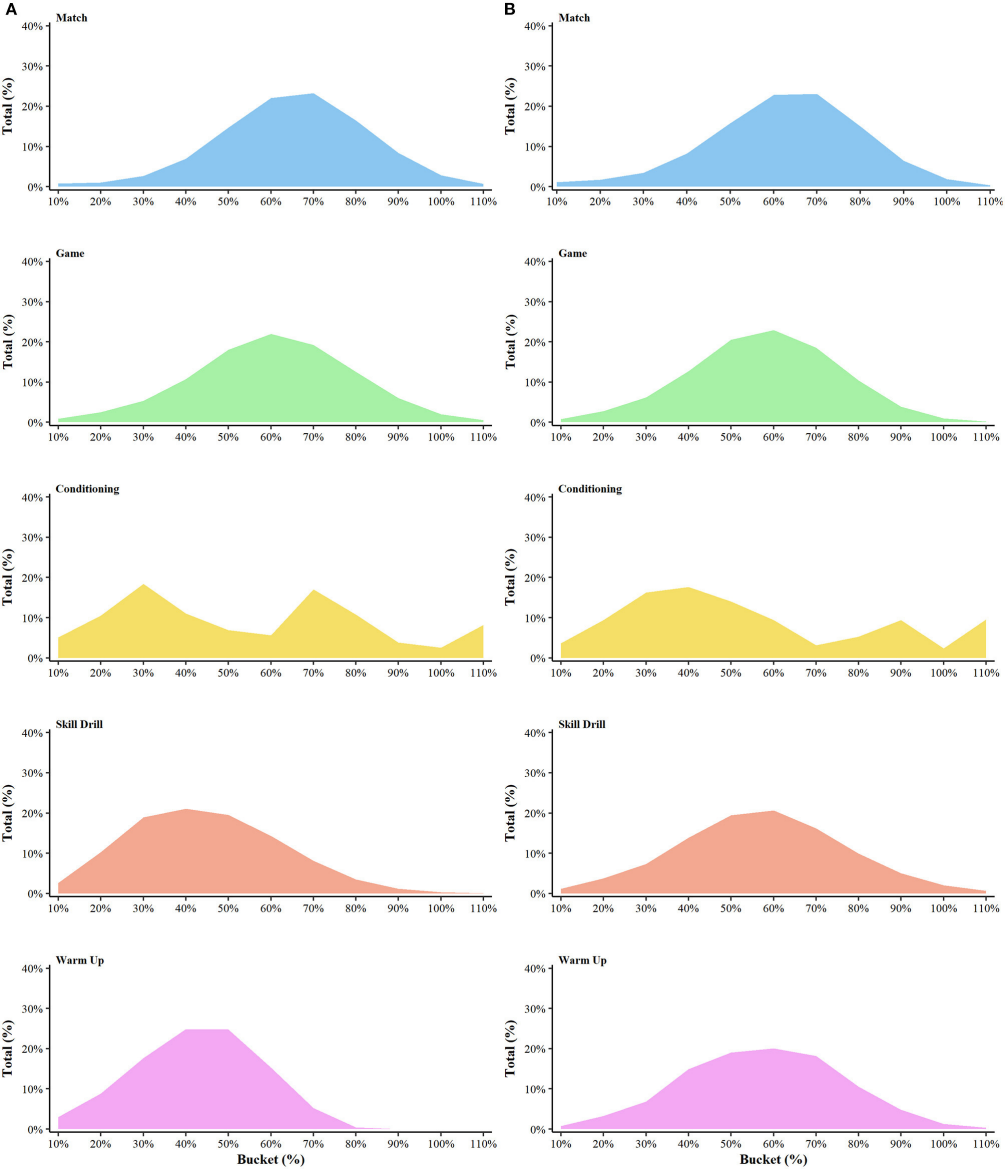
Distribution of distance **(A)** and impulse **(B)** for each drill type.

Figure 2 depicts the mean and individual weekly externally measured training volumes during training and matches across the pre-season and in-season phases (no statistical comparisons made). Table 2 provides the descriptive data (mean ± SD) of the volume and percentage of distance and impulse accumulated within each intensity bucket for each drill type. Figure 3 depicts the percentage distribution of distance (A) and impulse (B) within each intensity bucket for each drill type.

**Table 2:** Descriptive data (mean ± standard deviation) of the volume and percentage of distance and impulse accumulated within each intensity bucket for each drill type.

**Bucket**	**Variable**	**Match**		**Game**		**Conditioning**		**Skill drill**		**Warm up**	
		**Volume**	**Percentage**	**Volume**	**Percentage**	**Volume**	**Percentage**	**Volume**	**Percentage**	**Volume**	**Percentage**
<50%	Distance	666 ± 85	12 ± 1%	231 ± 43	20 ± 4%	81 ± 21	45 ± 18%	253 ± 57	53 ± 13%	319 ± 52	54 ± 8%
	Impulse	16625 ± 2266	15 ± 2%	5859 ± 1218	23 ± 4%	2710 ± 975	47 ± 15%	3903 ± 1020	26 ± 7%	4694 ± 895	26 ± 5%
50%	Distance	850 ± 334	15 ± 5%	229 ± 141	18 ± 9%	25 ± 29	7 ± 12% ^*^	120 ± 107	20 ± 15%	134 ± 63	24 ± 12% ^*^
	Impulse	18275 ± 6361	16 ± 6%	5652 ± 3878	21 ± 9%	761 ± 831	14 ± 18 ^*^	3054 ± 2579	19 ± 13%	3493 ± 2142	19 ± 10%
60%	Distance	1280 ± 392	22 ± 6%	295 ± 180	22 ± 9%	28 ± 56	6 ± 11% ^*^	106 ± 134	14 ± 14% ^*^	84 ± 57	15 ± 11% ^*^
	Impulse	26721 ± 6862	23 ± 5%	6295 ± 3854	23 ± 8%	546 ± 717	9 ± 16% ^*^	3368 ± 2712	21 ± 13%	3564 ± 2322	20 ± 11%
70%	Distance	1358 ± 370	23 ± 4%	279 ± 196	19 ± 9%	134 ± 250	17 ± 29 ^*^	72 ± 128	8 ± 11% ^*^	31 ± 43	5 ± 8% ^*^
	Impulse	27671 ± 9076	23 ± 5%	5028 ± 3350	19 ± 9%	331 ± 760	3 ± 7% ^*^	2838 ± 2657	16 ± 13%	3144 ± 1908	18 ± 11%
80%	Distance	971 ± 398	16 ± 5%	199 ± 200	13 ± 9%	87 ± 193	11 ± 23% ^*^	36 ± 89	3 ± 7% ^*^	5 ± 23	1 ± 2% ^*^
	Impulse	18426 ± 9230	15 ± 6%	2833 ± 2702	10 ± 9%	918 ± 2793	5 ± 14% ^*^	1862 ± 2357	10 ± 11%	1816 ± 1539	10 ± 9%
90%	Distance	497 ± 335	8 ± 5%	103 ± 152	6 ± 7%	37 ± 112	4 ± 12%	14 ± 49	1 ± 4% ^*^	3 ± 21	0 ± 2% ^*^
	Impulse	8040 ± 6076	6 ± 4%	1046 ± 1617	4 ± 6%	1699 ± 4323	9 ± 22%	984 ± 1795	5 ± 8%	864 ± 1036	5 ± 6%
100%	Distance	165 ± 159	3 ± 3%	36 ± 75	2 ± 4%	29 ± 84	3 ± 7%	4 ± 21	0 ± 2% ^*^	3 ± 21	0 ± 1% ^*^
	Impulse	2424 ± 3094	2 ± 2%	267 ± 677	1 ± 2%	480 ± 1557	2 ± 9%	389 ± 1096	2 ± 6%	236 ± 502	1 ± 3%
110%	Distance	44 ± 83	1 ± 1%	10 ± 36	1 ± 2%	115 ± 344	8 ± 21%	1 ± 8	0 ± 1%	25 ± 170	2 ± 10%
	Impulse	464 ± 982	0 ± 1%	55 ± 293	0 ± 1%	2929 ± 8657	10 ± 26%	132 ± 595	1 ± 3%	65 ± 298	0 ± 1%

Compared to matches, differences in the distribution of volume that are >0.6 × the smallest worthwhile difference are denoted by *. Only statistical comparisons 50% or above were made.

When examining the proportion of distance covered at different intensities, compared to matches, a higher proportion of distance was accumulated at 50% of the MM within matches when compared to conditioning (ES = 0.86; ±90% CL = 0.36), however when compared to warm ups, there was a lower distribution of distance within matches at 50% (1.14; ± 0.48). At 60% of the MM, a higher proportion of distance was accumulated in matches when compared to warm-ups (0.80; ± 0.34), conditioning (1.87; ± 0.79) and skill drills (0.73; ± 0.31). At 70% of MM, a higher proportion of distance was covered in matches compared to both skill drills (1.79; ± 0.76) and warm-ups (2.78; ± 1.18). Similarly, at 80% of MM, a higher proportion of distance was covered in matches compared to both skill drills (2.09; ± 0.88) and warm-ups (3.93; ± 1.66), as it was at 90% for skill drills (1.54; ± 0.65) and warm-ups (2.23; ± 0.94), and at 100% for skill drills (1.05; ± 0.45) and warm-ups (1.43; ± 0.61).

Regarding impulse, at 60% of MM, there was a higher proportion of impulse accumulated within matches compared to conditioning drills (1.06; ± 0.45), similarly at 70% (2.70; ± 1.14) and 80% (0.91; ± 0.38). There were no other substantial differences in the proportion of impulse between matches and drill types at each bucket.

When comparing the distribution of volume covered within each bucket for drill types between each phase, more distance was accumulated at 50% during competition when compared to general preparation (1.08; ± 0.45). There were no other differences between each phase of the season for each drill type.

## Discussion

This investigation provided novel evidence of the “field-based” training requirements across an AFLW season. In part (a), the weekly externally measured training loads (including match load) for various metrics were presented (Figure 2), quantifying the periodization of training volume across a season. This analysis demonstrated that AFLW athletes undergo higher training loads during the pre-season phase for most variables that were assessed, a finding that is in agreement with periodization principles of team sports (Bompa and Haff, 2015; Moreira et al., 2020). In part (b), the intensity of training drills and matches relative to previously established MMs was investigated, where the volume of distance and impulse accumulated within intensity buckets (50–110% of MM) for different drill types was determined (Figure 3; Table 2). This analysis demonstrated that a higher proportion of distance is accumulated at intensities between 70 and 110% of MM within matches compared to each drill type (except conditioning). Further, a higher proportion of impulse is accumulated between 60 and 80% MM within matches compared to conditioning, however no other differences were evident. Additionally, the results of the present study demonstrated that in warm-ups, more proportion of distance was accumulated at 50% of MM during the competition phase compared to general preparation phase of the competition. Together, the findings presented within this study provide practitioners with useful information relating to both the volume and intensity of workloads undertaken across a season. This novel analysis of training drills compared to matches can help ensure athletes are exposed to not only the intensity of matches, but also the volume covered across a range of intensities is comparable. This has important applications for practitioners particularly from a rehabilitation perspective in the return to play process, as ensuring athletes have undertaken high intensity training that is comparable to that within matches.

This is the first study to describe the externally measured weekly workloads undertaken across an AFLW season. As depicted in Figure 2, compared to in-season the pre-season phase demonstrated evidently higher (although not statistically compared) field-based training loads for each metric, particularly distance, high-speed running and acceleration load. This findings is in agreement with established training periodization recommendations for team sports (Bompa and Haff, 2015; Moreira et al., 2020). As the primary aim of the pre-season period is to maximize the physical and technical abilities of athletes in preparation for the preceding competition, this finding is common to that of other research (Ritchie et al., 2016; Moreira et al., 2020). Although this study did not examine internal loads, often this pre-season period is also characterized by higher internal load (i.e., session rating of perceived exertion) than the competition period (Rogalski et al., 2012), where emphasis is on recovery and rejuvenation between games to reduce the impact of fatigue on performance. Prior to this research, no such study within AFLW has investigated acceleration load, which is an important metric when considering the global acceleration/deceleration demands of team sports (Delaney et al., 2017b). As such, it is particularly important AFLW athletes are prepared to tolerate the extensive acceleration/deceleration demands of competition (Thornton et al., 2020).

No previous study has investigated the distribution of activity relative to MM within drills (as well as matches). This is a method that may help ensure certain training drills (e.g., match simulation, SSGs) have a comparable distribution of activity across a range of intensities when compared to matches (Table 2; Figure 3). Previous research has demonstrated the MM values of speed and acceleration across 1–10 min periods within AFL (Delaney et al., 2017a) and AFLW (Johnston et al., 2020), providing important information regarding the most intense periods of competition which is useful in the prescription of training. However, this “peak” of a game only occurs once within the game and fails to consider the volume of work performed at such an intensity (Johnston et al., 2020). A recent study (Johnston et al., 2020) investigated the distribution of activity relative to the 1-min MM intensity within AFL and rugby league, demonstrating that most activity is performed at ~60% of the MM, and for AFL, just 13% of distance was accumulated above 70% of the MM. Within this study, a higher proportion of distance was covered above 70% (51% and 3.1 km), although this was lower for impulse (46% and 57,025 kN·s^−1^). Interestingly, above 100% of the MM, there was minimal volume covered for both distance and impulse, reflecting the notion that covering large volumes at these intensities is not necessary to replicate the most demanding periods of competition in training (Johnston et al., 2020). Overall, these findings demonstrate that preparation of AFLW athletes should involve high-intensity skill-based drills (i.e., SSGs, match simulation) periodized within their training program, as these drills can expose athletes to periods of high-intensity work, whilst simultaneously developing the skill component (Weaving et al., 2017; Duthie et al., 2019; Johnston et al., 2019). It can be hypothesized that the capacity to perform at high intensities for sustained periods may have a tactical performance benefit, as this may permit athletes to physically out-perform their opponent, potentially resulting in a greater number of uncontested possessions, thus increased scoring potential.

In addition to investigating the distribution of activity within each drill type, this research compared these distributions to that of matches, to identify if certain drills involve similar physical demands. It was expected that games (i.e., SSG) display a comparable distribution of distance and impulse to that of competition, as games are used by practitioners as a tool to prepare and overload physical and tactical match demands (Duthie et al., 2019). This analysis demonstrated that at 60–100% of the MM, there was a higher proportion of distance within matches compared to conditioning, skill drills and warm-ups (Table 2; Figure 3). As AFLW is an intermittent sport (Clarke et al., 2018; Thornton et al., 2020), a large portion of activity is completed at lower intensities as demonstrated in this study, where 60% of the MM represents a mean speed of 123 m·min^−1^. As the intensity increased, for both distance and impulse there were minimal differences between the distribution of activity of drills and matches, showing that the distribution of intensity is alike that of matches. Interestingly, at any intensity bucket, there was no substantial difference in the distribution of distance and impulse within training games to that of matches. From a physical preparation perspective, this finding reflects that games (i.e., SSGs) are a useful tool in exposing athletes to the fluctuating intensities common to that of competition (Figure 2), whilst simultaneously developing technical abilities. This study also investigated whether the distribution of intensity within each drill type varied across each phase of the season, to identify if the purpose of each phase influenced the outcome of the intensity distribution within drills. Interestingly, there was only one substantial difference for warm-ups, where at 50% of the MM, a higher distribution was prevalent within competition compared to general preparation (1.08; ± 0.45). This finding may reflect the reduced intensity of warm-ups within competition, where a greater emphasis on recovery and rejuvenation is a key focus. It was expected that games and perhaps skill had a greater distribution of activity at higher intensities (i.e., 80–110%) within specific preparation compared to general preparation, as typically during this phase the aims of training drills is to mimic match scenarios. A likely cause of this not occurring within the present study is that this investigation was conducted within an inaugural season, where training was largely focused on developing tactical skills, where match roles were established and learning key concepts, therefore, drills may not have largely altered as the season progressed.

## Conclusions

Overall, this study aimed to provide an overview of the training undertaken across an AFLW season, where for a range of externally measured metrics, weekly training loads (also included match load) was summarized. This demonstrated the extensive workloads that are completed across a season by AFLW athletes, emphasizing the importance of well–planned, structured training programs. In addition, this study examined the training intensity relative to previously established MM, where the volume and distribution of volume (%) completed within intensity buckets (50–110%) was determined. These findings showed that the highest proportion of volume within matches is performed at ~60% of the MM for both distance and impulse, a consistent finding with other research. In comparing the distribution within each bucket of matches against different drill types, the distribution of distance is higher between 70 and 100% within matches, compared to each drill type (except conditioning), and a greater proportion of impulse is accumulated between 60 and 80% within matches compared to conditioning. This novel analysis can be used by practitioners to plan and guide training, providing an understanding of the volume of activity performed relative to the 1-min MM. Additionally, when comparing the distribution of activity across season phases, for warm-ups, more distance is accumulated at 50% of MM within competition compared to general preparation, reflecting the differing training aims of these phases.

## Data Availability Statement

The datasets used and analyzed during the current study will be made available by the corresponding author upon reasonable request.

## Ethics Statement

The studies involving human participants were reviewed and approved by Australian Catholic University Human Research Ethics Committee. The patients/participants provided their written informed consent to participate in this study.

## Author Contributions

HT had the original idea of the paper, performed statistical analyses, wrote the paper, and prepared the figures. CA collected the data and assisted in editing the manuscript. AR oversaw the planning and prescription of the training program and assisted in editing the manuscript. RJ, GD, and CM contributed to the idea of the paper, assisted in preparing the figures, and editing of the manuscript. All authors contributed to the article and approved the submitted version.

## Conflict of Interest

The authors declare that the research was conducted in the absence of any commercial or financial relationships that could be construed as a potential conflict of interest.
